# Supply chain resource scheduling optimization of e-commerce enterprises in international trade based on mobile edge computing

**DOI:** 10.7717/peerj-cs.1407

**Published:** 2023-06-06

**Authors:** Qingxia Dong, Nana Chen, Shuai Wang

**Affiliations:** 1 Shandong Vocational and Technical University of International Studies, Rizhao, Shandong, China; 2 Rizhao Marine Engineering Vocational College, Rizhao, Shandong, China

**Keywords:** MEC, International trade, Supply chain, Resource scheduling

## Abstract

The cross-border e-commerce supply chain network (CBESCN) has extensive geographical coverage, trade barriers and complexity of cross-border logistics issues, which makes its construction and development face many challenges. This article focuses on solving the operation optimisation problem of CBESCN under the background of the Internet of Things. A genetic algorithm constructed and solved the resource scheduling model of the supply chain of e-commerce enterprises in international trade. In addition, the mobile edge computing (MEC) optimisation scheme based on partial computation unloading is involved. The initial offload ratio is set and supply chain resources are allocated, then the remaining computing resources are distributed according to the server’s computing power. Finally, the offload is optimised according to the resource allocation. The experimental results show that time delay and cost adjustment strategies can improve the CBE supply chain’s comprehensive ability. The supply chain optimisation scheme proposed in this article can effectively use supply chain resources according to the requirements of computing tasks to reduce the total delay of task execution and the consumption of node computing.

## Introduction

Various countries have been impacted by the spread of the COVID-19 epidemic and the measures to prevent and control the outbreak have seriously affected international trade and investment activities, which has broken the regular operation of the existing supply chain and industrial chain. In addition, the sudden change in the global political situation has made the international market turbulent. In this context, China’s complete supply chain presents significant advantages and even plays a vital role in ensuring global supply, among which cross-border e-commerce has made essential contributions (Zhou, Wang & Li, 2022). Cross-border e-commerce supply chain network (CBESCN) is a new supply chain network derived from customs, countries, and regions. Albino, Izzo & Kühtz (2002) expand it into a global supply chain with raw materials, intermediate products, finished products and other elements, with a sales network as the link.

With the continuous development of international trade, the supply chain and inventory management of e-commerce enterprises have become more and more complicated. To improve efficiency and reduce costs, many e-commerce companies are looking for effective solutions (Wang, Jia & Schoenherr, 2020; Wang, Jia & Schoenherr, 2018). MEC technology provides a new solution that can help enterprises optimise supply chain and cost management, which realises real-time data processing and analysis by moving data processing and analysis to edge devices (Zhang, Cai & Xiao, 2021; Liu, Pan & Zhang, 2021). This benefits the supply chain and inventory management of e-commerce enterprises because it can help enterprises monitor the supply chain operation and the inventory changes in real time. By using MEC technology, e-commerce enterprises can control the supply chain and inventory more flexibly, thus improving efficiency and reducing costs. Various forms of computing-intensive data processing have emerged in large numbers, and massive traffic has seriously burdened the backbone network (Abbas, Zhang & Taherkordi, 2018). To avoid transmitting application data to the remote cloud, MEC deploys resources of computing, storage and other services to the network’s edge close to users, responds to users’ requests in time, and brings users a better experience (Nguyen & Dressler, 2020; Lin, Liao & Jin, 2019). Due to the limited computing and storage resources of mobile terminal devices, the high-performance computing requirements of these applications cannot be guaranteed. Computing offloading shortens the processing time by offloading some or all computing tasks to the edge server instead of the cloud server, which is an effective means to solve the problem of the insufficient computing power of mobile terminal devices and prolong the use time. How to realise collaborative computing between mobile terminal devices and edge servers is a hot issue in current research (Wangfi, Wang & Tan, 2020). Moreover, how to survive in the turbulent market environment, maintain the stability of the supply chain and comprehensively use CBE service capabilities and other means to meet customers’ needs, seek long-term development, and improve the operational performance of the supply chain has become the focus of CBE supply chain enterprises.

This article discusses the role of MEC optimisation in the supply chain and costs optimisation control of e-commerce enterprises in international trade. The main innovations are:

(1) The time scheduling optimisation model of CBESCN with time constraints is established, with the total cost, order completion time and CBE service provider’s satisfaction as the optimisation objectives;

(2) Analyse the influence of MEC on the supply chain and inventory management of e-commerce enterprises and how to optimise the node computing efficiency of the supply chain network using MEC.

## Related Works

### Supply chain network optimisation

The reasonable arrangement of CBESCN can enhance its reliability. In the node scale design of a supply chain network, Toro, Franco & Echeverri (2017) constructed a partition integer programming model considering inventory cost, drop shipment cost and equipment location cost, and determined the optimal quantity and location of warehouses and the optimal inventory strategy of warehouses and retailers. Moghaddam Kamran (2015) proposed a mixed integer linear programming model for designing on and reverse-flow supply chain networks. They verified the model’s feasibility through the European supply chain case.

In putting forward the optimisation goal, fuzzy optimisation, robust optimisation, topological network theory and system engineering are used to study some optimisation problems of CBESCN. Liu & Li (2020) took cross-border electronic commerce as the background. The research method discusses how to develop a set of corresponding technologies and methods under the blockchain background to achieve the traceability of products and transactions in supply chain management. Regarding material storage and distribution, especially for inventory control, which still needs to be optimised to minimise costs while maintaining high customer satisfaction, Yu, Hou & Li (2019) applies the basic ideas of the ant colony algorithm and fuzzy model to the supply chain inventory optimisation model. Yan, Zhou & Li (2021) pointed out that due to problems, such as explosive growth trends and high transportation costs in cross-border electronic commerce, it is urgent to study the supply chain from the perspectives of decision-making and coordination.

In the existing research on the joint optimisation of resource allocation and computing and unloading, the main consideration is the scenario of complete unloading. There are few types of research on partial unloading and resource joint optimisation; significantly, when the number of users increases, the number of computing increases, and the computing capacity of edge servers is limited. How to optimise the amount of computing data for unloading is the leading research content of this work.

### MEC

Much attention has been paid to improving the overall performance of the MEC system by offloading computation and jointly optimising various resources (Dai, Liu & Guo, 2019; Mukherjee et al., 2019; Jošilo & Dán, 2019; Eshraghi & Liang, 2019; Zhu, Chen & Chen, 2019). Dai, Liu & Guo (2019) built a layered edge cloud computing architecture for a scenario with a large amount of computing. Taking energy consumption of terminal equipment and task execution delay as optimisation objectives, a centralised computing unloading method based on the Lagrange multiplier method and an optimal resource allocation mechanism was proposed for mobile edge nodes. The computing and communication resources among multiple edge servers and between edge servers and cloud servers are jointly optimised. Quadratic programming with quadratic constraints obtains an approximate solution to ensure task delay (Mukherjee et al., 2019). Jošilo & Dán (2019) based on the Stackelberg game model, analysed how mobile devices used operators’ communication and computing resources independently and proposed a decentralised approximate computing unloading decision algorithm to minimise the task completion time of mobile terminal devices. Because the computing requirements are not fully known before the task is executed, Eshraghi & Liang (2019) proposed a method to jointly optimise the unloading decision to minimise the weighted sum of the average cost and cost changes. Zhu, Chen & Chen (2019) proposed a joint unloading decision and resource allocation algorithm based on deep learning, which comprehensively considered the optimisation of unloading decision, local CPU, bandwidth and external CPU occupancy to reduce task delay and equipment energy consumption. Gao, Zhang & Yu (2022) proposed a system overhead minimisation model for ultra-dense network scenarios, combining the unloading strategy, channel allocation and power allocation. Partial unloading can upload some tasks to the edge server for calculation under limited bandwidth and effectively use the computing resources of mobile devices and servers in parallel (Bozorgchenani, Tarchi & Corazza, 2018). Bi & Zhang (2018) studied the maximisation of the weighted sum computing rate in multi-user wireless edge computing networks with a binary computing offload strategy.

The scenario of complete offloading is mainly considered in the existing research on joint optimisation of resource allocation and computing offloading. In contrast, the research on joint optimisation of partial offloading and resources is less, especially on optimising the offloaded computing data when the number of users increases and the computing capacity of the edge server is limited.

## Resource Scheduling Model of CBESCN in International Trade

### Model construction

**Figure 1:**
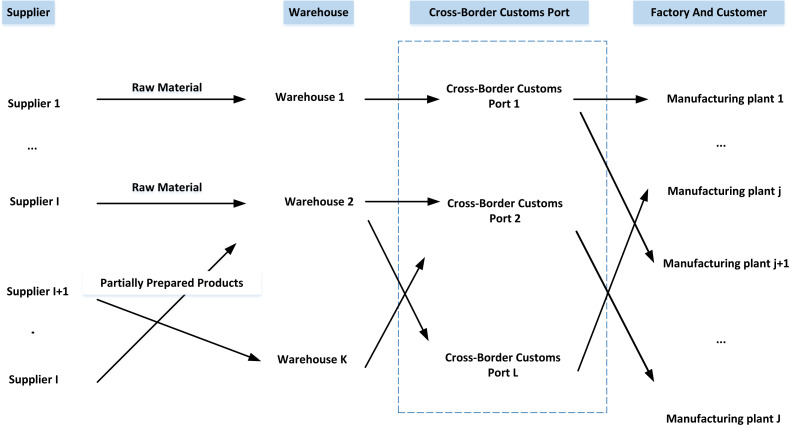
CBESCN structure diagram.

Consider a typical CBESCN spanning two different countries or regions, in which the network boundary is the supply chain transportation route, and the nodes are suppliers, warehouses, cross-border checkpoints, manufacturing plants and end customers in the logistics transportation route, as shown in Fig. 1.

Since the cross-border inspection point is located downstream of CBESCN, the warehouse delivery process should be emphasised. If unique warehouse picking and distribution operations are arranged to adapt to any particular situation, the chance of delay can be significantly reduced. Secondly, at the cross-border inspection point, the delivery of goods requires cross-border inspection procedures, including product inspection and verification of delivery documents. Diversifying the cross-border inspection process will lead to longer and longer transportation time and higher transportation costs. Given this, reducing the barrier effect of the cross-border supply chain that the delivered products meet the inspection is a fundamental rule.

This article constructs a resource scheduling model of the supply chain of e-commerce enterprises in international trade.

The objective function K1 represents the total cost in CBESCN. It consists of four parts: the total cost of all services in the supply chain, including conventional and other logistics services that are different from other logistics models. The second part is the time delay cost caused by the delay of the previous process due to the enterprise service process. The third part, the last process, is compressed by the enterprise service process, which leads to the time compression cost of the next process. The fourth part is the cost of sorting and packaging in overseas warehouses C_0_. In objective function K1, the delay of the previous process due to the enterprise service process leads to the time delay cost of the next process, which is realised in different cross-border enterprises. It can be expressed as

Total cost = Normal service cost + Delayed service cost + Compressing service cost + C_0_

(1) \begin{eqnarray*}\begin{array}{@{}ll@{}} \displaystyle {K}_{1}=&\displaystyle \sum _{\mathrm{m}=1}\,\sum _{\mathrm{n}=1}\, \left( {C}_{\mathrm{mn}}{T}_{\mathrm{mn}}+{C}_{\mathrm{mn}}^{{}^{{^{\prime}}}} \left\vert {T}_{\mathrm{mn}}^{{}^{{^{\prime}}}} \right\vert \right) +\sum _{\mathrm{m}=1}\, \left( {T}_{\mathrm{mn}}+{T}_{\mathrm{mn}}^{{}^{{^{\prime}}}}-{\mathrm{t}}_{\mathrm{m}} \right) {X}_{\mathrm{m}}^{{}^{{^{\prime}}}}\\ \displaystyle &\displaystyle +\sum _{\mathrm{m}=1}\, \left( {\mathrm{t}}_{\mathrm{m}}-{T}_{\mathrm{mn}}-{T}_{\mathrm{mn}}^{{}^{{^{\prime}}}} \right) {Y}_{\mathrm{m}}^{{}^{{^{\prime}}}}+{C}_{0}. \end{array}\end{eqnarray*}

The objective function K2, which consists of three parts, indicates that all logistics service orders in CBESCN should be completed as soon as possible. This article considers minimising the difference between expected and normal service time, extra service time and total time. That is, the expression is:

Time = expected service time - normal service time - extra service time + T_0_

(2) \begin{eqnarray*}{K}_{2}=\sum \left( {\mathrm{t}}_{\mathrm{m}}-{T}_{\mathrm{mn}}-{T}_{\mathrm{mn}}^{{}^{{^{\prime}}}} \right) +{\mathrm{T}}_{0}.\end{eqnarray*}

The objective function K3 indicates that the weighted satisfaction of all service provider enterprises can be maximised in the CBESCN. It consists of two parts: the first refers to the ratio of regular service cost to all average total costs generated by all service provider enterprises, representing satisfaction with the price. The other part is satisfied with service time. The second one is related to time, which refers to the ratio of the average operation time of service provider enterprises to the sum of standard time and extra time.

(3) \begin{eqnarray*}{K}_{3}=\sum _{m=1}\sum _{n=1} \frac{{T}_{\mathrm{mn}}{C}_{\mathrm{mn}}}{{T}_{\mathrm{mn}}{C}_{\mathrm{mn}}+{C}_{mn}^{{}^{{^{\prime}}}} \left\vert {T}_{\mathrm{ mn}}^{{}^{{^{\prime}}}} \right\vert } \left( \frac{{T}_{\mathrm{mn}}}{{T}_{\mathrm{mn}}+{T}_{\mathrm{mn}}^{{}^{{^{\prime}}}}} \right) {\mathrm{a}}_{\mathrm{mn}}.\end{eqnarray*}

Regarding constraints, the m-th service process can sustain the upper limit of time compression. This is a strong constraint because it is necessary to ensure a continuous running time link of the supply chain in the process of providing imported logistics services.

(4) \begin{eqnarray*}{\mathrm{t}}_{\mathrm{m}}-{T}_{\mathrm{mn}}-{T}_{\mathrm{mn}}^{{}^{{^{\prime}}}}\leq {T}_{\mathrm{ m}+1}^{{}^{{^{\prime}}}}.\end{eqnarray*}

The second constraint is that CBE service integrators put forward the completion time requirements for service orders of corresponding service provider enterprises.

(5) \begin{eqnarray*}{T}_{\mathrm{mn}}+{T}_{\mathrm{mn}}^{{}^{{^{\prime}}}}\leq {\mathrm{t}}_{\mathrm{ m}}+{\mathrm{b}}_{\mathrm{m}}Q{\mathrm{t}}_{\mathrm{m}}.\end{eqnarray*}

The third constraint is to ensure the satisfaction of each service provider enterprise, which must be higher than their acceptable lower limit.

(6) \begin{eqnarray*} \frac{{T}_{\mathrm{mn}}{C}_{\mathrm{mn}}}{{T}_{\mathrm{mn}}{C}_{\mathrm{mn}}+{C}_{mn}^{{}^{{^{\prime}}}}\mid {T}_{\mathrm{ mn}}^{{}^{{^{\prime}}}}} \left( \frac{{T}_{\mathrm{mn}}}{{T}_{\mathrm{mn}}+{T}_{\mathrm{mn}}^{{}^{{^{\prime}}}}} \right) \geq {S}_{\mathrm{mn}}^{{}^{{^{\prime}}}}.\end{eqnarray*}

In terms of time, cross-border e-commerce logistics service integrators tend to follow customised time requirements and set corresponding requirements related to order completion time for service providers. The satisfaction of each service provider will directly determine the quality of the order and the likelihood of completion.

### Model solving

This article selects the linear weighting method to solve the CBE time scheduling optimisation model, and the above model is transformed into a single objective programming problem. The linear weighting method can make the interaction of each evaluation index get linear compensation and ensure the fairness of the comprehensive evaluation index. Firstly, the data is imported, then the multiple linear regression is carried out on the data, and the regression is carried out on all variables. The stepwise regression can also be used for factor screening, and the model after the preferred factor can be obtained and the objective function is finally solved.

In this article, the cost adjustment factor d is introduced into the model, which represents the relationship of cost increase in this case. d > 1 to express the limit of cost increase. Because I set a new constraint, the original time scheduling optimisation model becomes a goal-planning problem about time and satisfaction.

(7) \begin{eqnarray*}{K}_{1}\lt {K}_{1}^{\min \nolimits }\ast \mathrm{d}.\end{eqnarray*}

The model constructed in this article has three objective functions and four constraints. We combine the specific problems and make a choice analysis, which will help solve the problems and improve efficiency. We introduce *V*_2_ and *V*_3_, and make them add up to 1. Objective function K_2_ And objective function K_3_ respectively means that the service order is completed on time as much as possible, and the satisfaction of the service provider is maximised. This article needs to standardise them. Therefore, K_2_ and K_3_ Divide by the maximum possible value and subtract from the corresponding results. t the comprehensive objective function K can be obtained as follows:

(8) \begin{eqnarray*}K={V}_{3} \frac{{K}_{3}}{m{K}_{3}} -{\mathrm{V }}_{2} \frac{{K}_{2}}{m{K}_{2}} .\end{eqnarray*}

## MEC Based on Partial Computation Unloading

### Problem description

MEC is used to optimise the solution process proposed in ‘Resource scheduling model of CBESCN in international trade’ to improve the efficiency of node calculation. It is assumed that the MEC system knows relevant information such as channel gain, calculated data volume, calculated energy consumption, *etc*., to determine the amount of data unloaded by each user and the allocated channels and computing resources, thereby reducing the calculation delay and energy consumption of users.

Each supply chain node *k* contains a computation-intensive task with limited delay to complete, and the maximum delay of the task is ${T}_{k}^{\max }$, the maximum energy consumption is ${E}_{k}^{\max }$, the data size of the task is *S*_*k*_. The CPU cycle required to compute each bit on the client side is *C*_*k*_ . When users *k* When the computing power of cannot meet the requirements of task delay or energy consumption, all or part of the task can be unloaded to the edge server of the base station, and the unloading ratio is *λ*_*k*_ ∈ [0, 1] . Therefore, the time that the task runs locally is ${t}_{k}^{\text{local}}$, expressed as

(9) \begin{eqnarray*}{t}_{k}^{\text{local}}= \left( 1-{\lambda }_{k} \right) {S}_{k}{C}_{k}/{f}_{k}^{0}\end{eqnarray*}

where ${f}_{k}^{0}$ represents the computing power of user *k*.

The energy consumption per CPU cycle of node *k* is ${e}_{k}^{0}$. When some tasks are executed locally, the energy consumption is

(10) \begin{eqnarray*}{e}_{k}^{\text{local}}= \left( 1-{\lambda }_{k} \right) {S}_{k}{C}_{k}{e}_{k}^{0}.\end{eqnarray*}

The user unloads a part of the task to the edge server, downloads the calculation results from the edge server, and calculates the remaining tasks at the same time. Because the calculation result is very small, the download time of the calculation result is not considered. Task execution time only considers unloading task upload time ${t}_{k}^{\text{off}}$, edge server execution time ${t}_{k}^{svv}$ and the local execution time of the remaining tasks ${t}_{k}^{\text{local}}$. Because of the parallelism of local computing and edge server computing, the total computing time to complete the task is ${t}_{k}^{\text{total}}$, expressed as:

(11) \begin{eqnarray*}{t}_{k}^{\text{total}}=m \left( {t}_{k}^{\text{local}},{t}_{k}^{\text{off}}+{t}_{k}^{svv} \right) .\end{eqnarray*}

Multi-user partial unloading with resource allocation under time delay and energy consumption constraints in MEC is considered as an optimisation problem, which is to minimise the total delay of task completion. The goal of problem optimisation is expressed as:

(12) \begin{eqnarray*}\min _{\lambda ,\delta ,p}\,\sum _{\mathrm{k}=1}^{N}m \left( \frac{ \left( 1-{\lambda }_{k} \right) {\mathrm{S}}_{k}{C}_{k}}{{f}_{k}^{0}} ,m \left( \frac{{S}_{k}^{i,off}}{{r}_{k}^{i}} \right) + \frac{{\lambda }_{k}{S}_{k}{C}^{svv}}{{f}_{k}^{svv}} \right) \end{eqnarray*}

### Partial computation unloading

The optimisation goal of the system is to minimise the total delay in task completion. As can be seen from Formula (12), this optimisation problem mainly involves the unloading ratio and system resource allocation (Li, Li & Zhou, 2021). Therefore, this article decomposes the optimisation problem into two aspects: unloading ratio setting and resource allocation. Firstly, according to each user’s time delay and energy consumption limit, an initial unloading ratio is given, then a low-complexity joint allocation strategy of channel and server computing resources is proposed. Finally, the unloading ratio is updated according to the currently available resources.

Firstly, the computing resources are initially allocated according to the following delay constraints: on the one hand, the unloading delay of some data must not exceed the local computing delay, that is, ${t}_{k}^{off}\lt {t}_{k}^{\text{local}}$; On the other hand, the calculation delay of the whole task shall not exceed the total delay limit, that is ${t}_{k}^{\text{total}}\lt {T}_{k}^{\max }$ . The second allocation of computing resources is the allocation of the remaining available computing units, where the user’s computing rate difference is defined here as the objective function *σ*, that is, a computing resource is allocated to user *k*, and the computing rate difference before and after the computing resource is allocated is expressed as

(13) \begin{eqnarray*}\sigma = \frac{{S}_{k}}{{t}_{k}^{\text{local}}+{t}_{k}^{off}+{t}_{k}^{svv+1}} - \frac{{S}_{k}}{{t}_{k}^{\text{local}}+{t}_{k}^{off}+{t}_{k}^{svv}} \end{eqnarray*}

where ${t}_{k}^{sv+1}$ is the computing time of the server after the server assigns a new cell to the user. Comparing the calculation rate difference between each user before and after obtaining the calculation unit, and assigning a calculation unit to the user who can gain the greatest benefit by adding the calculation resources for the user so that *k*^∗^ = argmax(*σ*).

After completing the initial allocation of resources for each supply chain node, the system determines the available computing resources of the edge server. It updates the user’s unloading ratio so that more computing tasks of mobile users are unloaded to the edge server for execution, and the computing delay is reduced. When the calculation time of the local task and partial unloading calculation time is balanced, the delay reaches the minimum. The user’s updated unloading ratio can be obtained from Eq. (13).

(14) \begin{eqnarray*}{\lambda }_{k}= \left( \frac{{S}_{k}}{{f}_{k}^{0}} -m \left( \frac{{S}_{k}^{i,\text{off}}}{{r}_{k}^{i}} \right) \right) /{S}_{k} \left( \frac{1}{{f}_{k}^{sv}} + \frac{1}{{f}_{k}^{0}} \right) .\end{eqnarray*}

Because the computing resources are dynamically changing when Eq. (14) is less than 0, it means that the resource conditions cannot meet the task requirements, and the unloading ratio is 0; When Eq. (14) is greater than 1, it means that the resources are sufficient to completely unload the task execution, and the unloading ratio is 1.

## Experiment and Analysis

### experimental settings

Take the optimisation of the CBESCN location of a CBE enterprise as an example to analyse. To meet the decentralised needs of consumers and reduce the logistics cost of the whole cross-border supply chain, the optimisation of terminal distribution path selection from network nodes such as overseas warehouses to demanders is also a common decision variable in the location scheme. MEC simulation scenario considers a base station and multiple nodes, and the base station deploys edge servers. The radius of the coverage area of the base station is 500 m, and the supply chain nodes are randomly distributed in this area.

### Total time solution for service providers

**Figure 2:**
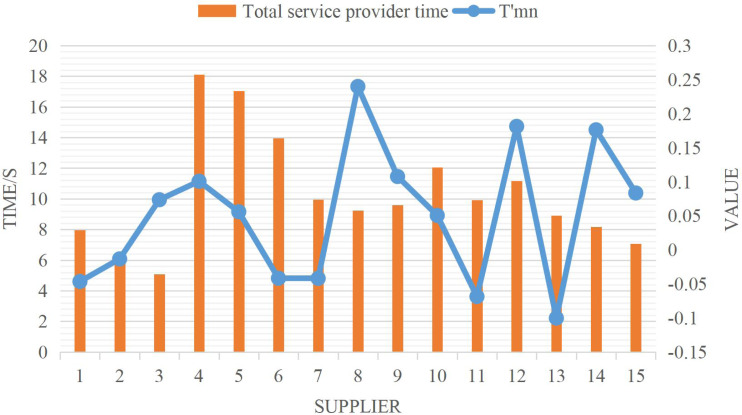
Model solution results.

Assuming that the recombinant inbred lines are 200 and the genetic algebra is 300 when the time adjustment coefficient is *Q* = 1 and the cost adjustment coefficient is *d* = 1.15, the calculation result of the objective function K is equal to 0.6612. The time parameters of each service provider in each service process are shown in Fig. 2.

From the results of an example, in completing the CBESCN, among the 15 service provider enterprises, six completed time-compressed orders and nine completed time-delayed orders. It shows that when different functional service providers face service orders, CBE service integrators will delay or compress the service time in different service processes, although the compressed time will be more responsive to customer needs.

**Figure 3:**
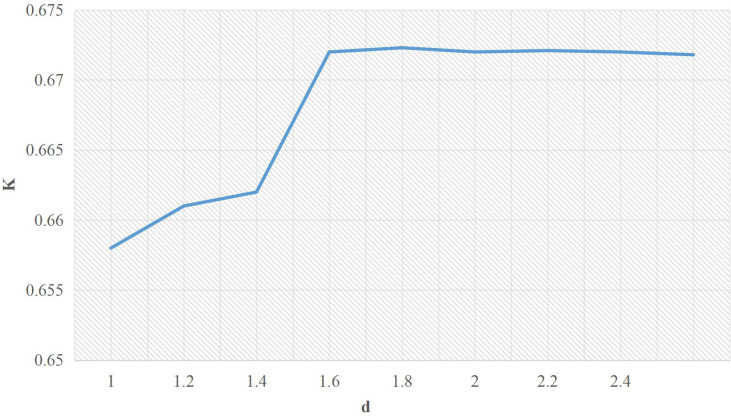
Range of *K* value.

Figure 3 shows the range of the *K* value. With the cost adjustment coefficient increase, *K* also increases, which is stable at 0.6710. It means that when the cost adjustment coefficient of CBE service integrators increases, the comprehensive level of CBESCN is also improved.

The above analysis and comparison show that the time adjustment coefficient of the CBE service integrator can be divided into two stages. When the time is compressed to the time delay, the comprehensive level of CBESCN increases with the increase of the time adjustment coefficient. However, after reaching a specific value, the comprehensive level of CbesCN will be stable, and the effect will be relatively apparent. Both the time delay strategy and the cost adjustment strategy can somewhat improve the comprehensive capability of CBESCN. However, the former works better.

### MEC optimisation results

The proposed method is simulated by Python and compared with local computing, complete and binary unloading (Bi & Zhang, 2018). Local computing means that all computing tasks are performed on the mobile terminal. Full uninstall means all computing tasks are uninstalled to the edge server for execution. Binary unloading refers to choosing a local computing or unloading mode by comparing local computing and unloading computing rates.

**Figure 4:**
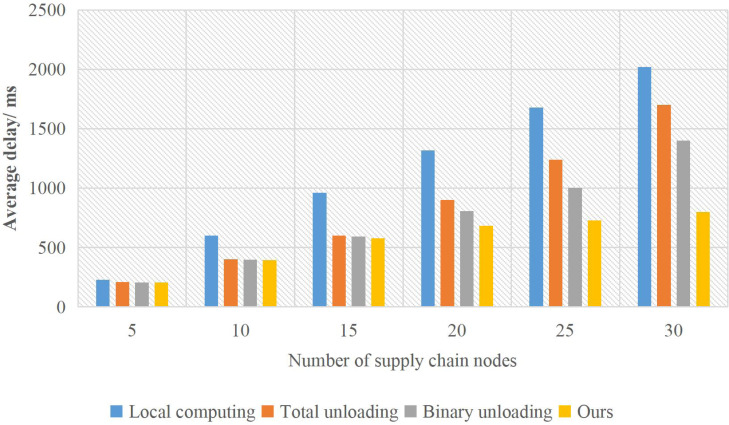
The influence of the number of users on the delay of supply chain nodes.

The influence of the number of nodes on the total delay is shown in Fig. 4.

 Because all tasks of local computing run on user terminals, the total delay increases linearly with the number of users. Complete unloading and unloading all tasks to the server for calculation. The delay of the server is lower when the number of stages is small. The computing resources allocated to each node decrease when the number of nodes increases. The total delay increases faster than when the number of nodes is small, but it is still lower than the local calculation. The adopted method is partial unloading. When the number of nodes is large, the competition for supply chain resources increases and the computing pressure of the server increases. Rational use of server resource allocation to optimise the number of task unloading can maximise the use of server computing resources, thus reducing the time delay of task completion.

**Figure 5:**
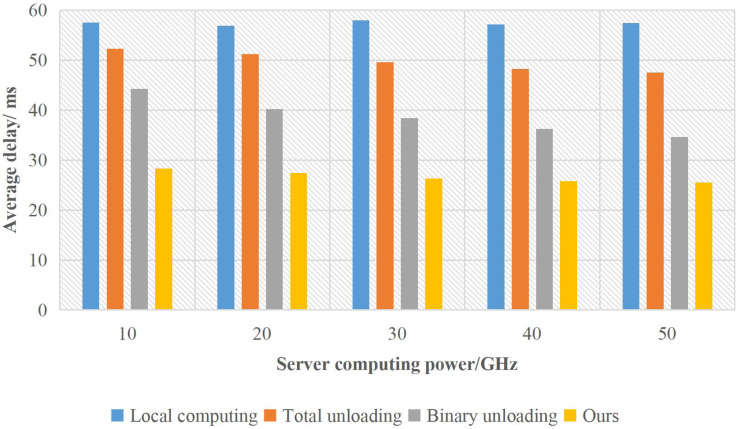
The influence of server computing power on the delay of supply chain nodes.

The influence of server computing power on the average delay of nodes is shown in Fig. 5.

Because local computing has nothing to do with the server, the average delay remains unchanged. The time delay of the complete uninstall method is only affected by network transmission and server computing power. With the increase in server computing power, the average time delay of users decreases significantly. In binary unloading, with the improvement of server computing power, the average delay of the system shows a slow decline trend. With the increasing capacity of the server, due to the limitation of network resources, the time delay gradually approaches the complete unloading mode. In this method, some tasks are transmitted to the server, and the user terminal and the server execute in parallel. Because the allocation of server computing resources is considered, the average delay of this method is low. When the server’s computing power reaches a certain level, the impact on the average delay of users mainly comes from network transmission.

## Discussion

Cross-border e-commerce service integrators should reasonably use time schedules to solve optimisation problems, minimise unnecessary demand for time compression, and prevent a sharp decline in the comprehensive level of the cross-border e-commerce logistics service supply chain. If it is necessary to improve the complete ability of cross-border e-commerce logistics service supply chain, cross-border e-commerce logistics service integrators should give priority to using the delay time strategy to analyse the influential factors of the cross-border supply chain network’s robustness, sources of uncertainty and reliability, to form a more comprehensive and systematic cross-border supply chain network system. This model provides theoretical support for quantitatively evaluating cross-border supply chain networks, node location, network design and other optimisation problems. The model is suitable for determining the separation point of customer orders under the background of mass customisation.

## Conclusion

Based on the study of CBESCN in many aspects, an optimisation model with CBESCN characteristics in the corresponding mode is constructed. Through the joint allocation of node equipment computing resources, supply chain resources and edge server computing resources, a method of partial computation unloading node computing intensive task to edge server is proposed. When different functional logistics service providers face service orders, enterprises delay or compress the service time in other service processes. This result is reflected in the increase of the cost coefficient; the objective function also increases and finally tends to be stable at 0.6710. In addition, this method transmits some tasks to the server, and the user terminal and the server execute them in parallel. Because the allocation of server computing resources is considered, the average delay of this method is low. Although it is helpful to provide new ideas for the theoretical development of CBESCN and its service supply chain, in this experiment, the information asymmetry brought from the perspective of service providers is not considered in the setting of the scheduling model, and the common GA algorithm is only used to solve the model. Our future research direction is how to use deep learning models further to optimise the operational efficiency of supply chain networks.

##  Supplemental Information

10.7717/peerj-cs.1407/supp-1 Supplemental Information 1 Dataset

10.7717/peerj-cs.1407/supp-2 Supplemental Information 2 Codes

## Additional Information and Declarations

### Data Availability

The following information was supplied regarding data availability: The data and code are available in the Supplementary Files.
